# Prognostic value of Ki-67 immunolabelling in primary operable breast cancer.

**DOI:** 10.1038/bjc.1993.389

**Published:** 1993-09

**Authors:** M. Railo, S. Nordling, K. von Boguslawsky, M. Leivonen, L. Kyllönen, K. von Smitten

**Affiliations:** Helsinki University Central Hospital, Finland.

## Abstract

The prognostic value of Ki-67 immunohistochemical labelling was evaluated in 327 operable primary carcinomas of the breast. The follow-up time was up to 4 years (mean 2.7 years). The disease-free survival in Ki-67 positive patients was shorter than in Ki-67 negative patients (P < 0.005). By combining the Ki-67 expression with ER receptors and stage, subgroups with a different disease-free survival were identified. In stage II patients there was a significant difference (P < 0.005) in disease-free survival between Ki-67 positive/ER negative and Ki-67 negative/ER positive patients. In node negative patients there was no such difference. The disease-free survival according to different prognostic factors, stage, ER and node status, were separately examined using a Cox's proportional hazards model. ER (P < 0.0001), the Ki-67 (P < 0.02), tumour size (P < 0.0001) and nodal status (P < 0.006) were independent prognostic factors. We conclude that the potential value of Ki-67 labelling for prognostic evaluation of patients with breast carcinoma is good.


Br. J. Cancer (1993), 68, 579–583 © Macmillan Press Ltd., 1993

### Prognostic value of Ki-67 immunolabelling in primary operable breast cancer

M. Railo[1], S. Nordling[2], K. von Boguslawsky[2], M. Leivonen[1], L. Kyllönen[1] & K. von Smitten[1]

[1]*Helsinki University Central Hospital and* [2]*Department of Pathology, University of Helsinki, Finland.*

**Summary** The prognostic value of Ki-67 immunohistochemical labelling was evaluated in 327 operable primary carcinomas of the breast. The follow-up time was up to 4 years (mean 2.7 years). The disease-free survival in Ki-67 positive patients was shorter than in Ki-67 negative patients ($P < 0.005$). By combining the Ki-67 expression with ER receptors and stage, subgroups with a different disease-free survival were identified. In stage II patients there was a significant difference ($P < 0.005$) in disease-free survival between Ki-67 positive/ER negative and Ki-67 negative/ER positive patients. In node negative patients there was no such difference. The disease-free survival according to different prognostic factors, stage, ER and node status, were separately examined using a Cox's proportional hazards model. ER ($P < 0.0001$), the Ki-67 ($P < 0.02$), tumour size ($P < 0.0001$) and nodal status ($P < 0.006$) were independent prognostic factors. We conclude that the potential value of Ki-67 labelling for prognostic evaluation of patients with breast carcinoma is good.

The increasing number of options for the treatment of breast carcinoma has made the prognostic evaluation of the disease even more important. The histologic criteria for grading of the carcinoma into poorly differentiated and well differentiated ones was established already in 1957 (Bloom & Richardson, 1957). Patients with poorly differentiated tumours have a shorter relapse-free survival than those with differentiated ones. Node positivity also decreases the disease-free survival time (Du-Toit *et al.*, 1990; Fisher *et al.*, 1983). Methods of $^3$H Thymidine incorporation (Silvestrini *et al.*, 1974) and flow cytometry (Kallioniemi *et al.*, 1987) are well established but complex procedures giving prognostic information. It is desirable to find easier methods for the prognostic evaluation of breast carcinoma. In this respect the introduction of a mouse monoclonal antibody Ki-67 by Gerdes *et al.* (1983) simplified the measurement of proliferative activity in breast carcinoma tissue. The monoclonal antibody labels a still unknown nuclear antigen in proliferating cells. Cell nuclei in the resting stage (GO) are not stained. Ki-67 immunoreactivity has been studied in several types of carcinoma, e.g. in breast carcinoma (Barnard *et al.*, 1987; Gasparini *et al.*, 1992; Locker *et al.*, 1992), in bladder carcinoma (Fontana *et al.*, 1992), in carcinoma of the prostate (Thompson *et al.*, 1992) and in colon carcinoma (Porschen *et al.*, 1991). In this study the prognostic value of Ki-67 immunohistochemistry is evaluated by a follow-up of 327 patients with primary breast carcinomas operated between 1988 and 1990.

### Patients and methods

A total of 327 patients operated for primary breast carcinoma between January 1988 and April 1990 at the II and IV Departments of Surgery of the Helsinki University Central Hospital were included in the study. Patients with a metachronous breast carcinoma less than 5 years previously were excluded as it is not possible to determine the origin of possible metastases. Nine synchronous breast carcinomas and two carcinomas, which had metastasised were included in the series. In patients with synchronous bilateral breast carcinoma, the statistical analysis was based on the breast with the more severe stage. The mean age of the patients was 57 years (range 28–86 years) and the mean follow-up time was 2.7 years with a maximum of 4 years. The p-TNM classification was done according to the pathologist's report and following the international classification system (TNM Atlas, 1992). In the graphic survival representation N1 and N2 patients were classified together because of the low number of N2 patients (10). Patients with T1 tumours, were usually treated with local resection only, unless the patient preferred mastectomy. A local resection was done in 15% of T2 carcinomas.

### *Tissue preparation*

The breast specimens were laid on ice and frozen in liquid nitrogen within 45 min. Sections for Ki-67 labelling, were stored at −20°. The acetone-fixed, air-dried sections were incubated for 60 min at room temperature with the primary Ki-67-antiserum (Dakopatts®) diluted 1:10. The rest of the staining procedure was performed using the avidin-biotin method (ABC-Kit, Vector Laboratories®, Burlingame, California). The result was visualised using AEC as chromogen. Finally the sections were lightly counterstained with Mayer's haematoxylin. For the immunohistochemical demonstration of ER frozen sections were stained with the ERICA kit (Abbott Laboratories®, Chicago, Illinois) according to the instructions of the manufacturer.

All 327 breast carcinomas were stained for Ki-67, and 281 tumours were also successfully stained for ER. The cut-off points for the Ki-67 and ER grading were arbitrarily chosen at the beginning of the study before the outcome of the carcinoma was known. The Ki-67 was graded as follows: Ki-67 negative: at the most, rare nuclear staining. Ki-67 weakly positive (+): about 1–2% nuclear staining. Ki-67 moderately positive (++): 3–10% nuclear staining. Ki-67 strongly positive (+++) > 10% nuclei stained. For the ER grade determination, ER up to 10% was scored as negative, 10–40% weakly positive, 40–70% moderately positive and over 70% strongly positive. The percentage of Ki-67 and ER positive cells was estimated by the same pathologist at the time of the operation from at least five randomly chosen medium power microscopic fields containing at least 2,000 cells. The Ki-67 was also independently scored by a biologist without knowledge of the outcome of the disease or of the score assessed by the primary observer. Metastatic disease was confirmed by radiological and clinical examination.

Ki-67 status was correlated to other prognostic factors: ER content, stage, tumour differentiation and nodal status. The relationship between disease-free survival and Ki-67 status, p-TNM, ER content and stage was also analysed.

### *Statistical methods*

The disease-free survival was determined using a life table analysis with a product limit method on all patients. The correlation analysis was done with the logrank-chi-square

Correspondence: Mikael Railo, Maria Hospital, Lapinlahdenkatu 16, SF-00180 Helsinki, Finland.
Received 14 April 1992; and in revised form 21 April 1993.

method. Multivariate analysis including p-TNM, and immunohistochemical determination of ER and Ki-67 was performed by the Cox's proportional hazards model.

### Results

Most tumours were ductal carcinomas (78%). Forty-one percent were stage I and 49% were stage II carcinomas. A breast resection was done in 46% of the patients with stage I and in 15% of those with stage II carcinomas. The overall resection rate was 61%. Most patients (137) were followed up to 3 years and 72 patients up to 4 years. The mean follow-up time was 2.7 years.

The disease-free survival (DFS) was significantly longer in node negative than in node positive patients ($P<0.005$) (Figure 1a), and the same applied to oestrogen receptor (ER) positive *vs* negative patients ($P<0.005$) (Figure 1b). The DFS decreased with increasing stage ($P<0.025$) (Figure 1c). The DFS according to different prognostic factors, stage, oestrogen receptor and node status, were separately examined using a Cox's proportional hazards model showing that oestrogen receptor ($P<0.0001$) and Ki-67 status ($P<0.02$) as well as tumour size ($P<0.0001$) and nodal status ($P<0.006$) were independent prognostic factors (Table I).

**Table I** Independent prognostic factors in breast carcinoma

| *Parameter* | *P-value* |
|---|---|
| Ki-67 | <0.02 |
| Oestrogen receptor | <0.0001 |
| Tumour size | <0.0001 |
| Nodal status | <0.006 |

**Figure 1** **a**, Disease-free survival substratified by nodale status. Node positivity was associated with worse prognosis ($P<0.005$). **b**, The disease-free survival substratified by ER. The ER negative subgroup had the worst prognosis. ($P<0.005$). **c**, the disease-free survival substratified by stage.

The overall patient survival according to Ki-67 expression showed no significant differences between the different Ki-67 groups (Figure 2a). On the other hand, there was a difference in the disease-free survival between Ki-67 negative and strongly positives (+ + +) ($\chi^2 = 17.3$, $P < 0.005$) (Figure 2b). There was no difference in the Ki-67 results obtained by the two independent observers. The correlation with DFS was the same. There were only small differences in the scores (Figure 3).

Neither the distribution of the Ki-67 expression according to the stage, nor the distribution of Ki-67 expression within the different groups of oestrogen expression showed any significant differences (data not shown). Likewise, the distribution of the Ki-67 expression according to the nodal status did not show statistically significant differences (data not shown). The histologic grading of the specimens was not done routinely, but to evaluate the correlation between Ki-67 and grade, a randomly chosen group of 40 specimens was graded. There was a positive correlation between Ki-67 labelling and tumour grade ($P = 0.007$).

If the patients were divided into groups according to the Ki-67 and ER expression to form different subgroups with potentially different risks (Figure 4), patients with Ki-67 positive and ER negative tumours had the shortest DFS ($P < 0.05$). Patients with Ki-67 negative and ER positive tumours had the longest DFS. The DFS was also significantly shorter for Ki-67 positive and ER positive tumours as compared to Ki-67 negative and ER positive tumours (Figure 4).

In an attempt to identify those stage I patients with poor prognosis, the stage I patients with ER negative and Ki-67 positive tumours were compared with those with ER positive and Ki-67 negative tumours. The difference in DFS between these groups was not significant, probably due to a too short follow-up time. On the other hand, in stage II tumours there was a significant difference in the disease-free survival when the same comparison was made for stage II tumours ($\chi^2 = 12.8$ $P < 0.005$), (Figure 5).

**Figure 3** Difference in Ki-67 score between two observers.

### Discussion

Because of the short follow-up period of the patients we chose to focus on the disease-free survival. Our study suggests that determination of the proliferative activity of breast carcinoma by immunohistochemical staining with the monoclonal antibody Ki-67 provides a useful prognostic indicator fo the relapse-free period in patients with breast carcinoma. The data suggest that if Ki-67 labelling is used alone as a prognostic factor a useful cut-off point delineating a high-risk group is 10% labelling nuclei, that is 'strongly positive' in our study.

We found that patients with Ki-67 positive, ER negative tumours had a clearly shorter disease-free survival than other combinations of these two parameters, and it may be relevant to routinely combine the results of these two analyses in the pathologist's report. The histologic grading

**Figure 2** **a**, Overall survival substratified by KI-67. No statistical differences. **b**, Disease-free survival analysis substratified by KI-67. The Ki-67 strongly positive group had the worst prognosis.

**Figure 4** Disease-free survival analysis substratified by KI-67 and ER. The Ki-67 positive ER negative subgroup had the worst prognosis ($P < 0.005$).

**Figure 5** Disease-free survival analysis, substratified by risk groups according to stage, ER and KI-67. In stage II patients, KI-67 positive and ER negative patients have the worst prognosis ($P < 0.005$).

of the tumours in this series also showed a positive correlation to Ki-67 labelling ($P = 0.007$) in agreement with previous reports (Barnard *et al.*, 1987; Gasparini *et al.*, 1992; Locker *et al.*, 1992; Weikel *et al.*, 1991).

From a practical point of view, one of the most important goals is to identify stage I patients with a poor risk (about 10% of the total) who might benefit from some type of adjuvant therapy. We failed to distinguish a subgroup of such poor risk patients in stage I (ER negative/Ki-67 positive), perhaps because of the small number of patients and the relatively short follow-up time of a maximum of 4 years. Neither has it been possible to single out those node negative patients, which will get a relapse using the EGFR expression, which is closely linked to cell proliferation activity (Toi *et al.*, 1991). Similar results for early relapses in node negative patients have been obtained with her/neu oncogene (Slamon & Clark, 1988). Cathepsin D has also failed to predict disease-free survival in node negative patients, while its predictive value was considerably strengthened when combined with ER-determinations (Granata *et al.*, 1991). Long term follow-up studies on stage I and node negative patients show that a recurrence can occur decades after the initial diagnosis. Those new methods which cannot be applied to fixed archivel tissue still need some time to show their value. In stage II tumours, including also node negative patients, there was a significant difference in disease free survival between Ki-67 positive/ER negative and ER positive/Ki-67 negative ($P < 0.005$). O'Reilly has described an even more specific subdivision of patients with different prognosis using the S-phase fraction (SPF) and tumour size (O'Reilly *et al.*, 1990). According to these results patients with tumour greater than 1 cm with an SPF < 10% have a 5-year relapse-free survival of 78%, compared to 52% in tumours of the same size with an SPF > 10%. The higher prognostic value of SPF compared to Ki-67 might be due to the longer follow up time (median 8 years). The SPF value is also higher in aneuploid tumours than in diploid tumours (O'Reilly, *et al.*, 1990) which might give SPF a better discriminatory power than Ki-67. On the other hand, the Ki-67 scores are much higher in aneuploid carcinomas than in diploid ones (Gasparini *et al.*, 1991).

In conclusion, despite the relatively short follow-up period of this study, our data suggest the potential value of Ki-67 labelling as a relatively simple and inexpensive method for prognostic evaluation of patients with breast carcinoma. The value of the method is enhanced if the result in the pathologist's report is given in conjunction with the immunohistochemical evaluation of oestrogen receptors. Until recently the study of Ki-67 proliferation antigen has been restricted to such cases, where fresh or frozen tissue is available. Recently new antibodies have been developed which also detect the Ki-67 antigen in paraffin embedded tissues (Cattoretti *et al.*, 1992).

This work was supported by grants from the Finnish Cancer Foundation and Finska Läkaresällskapet.

### References

BARNARD, N.J., HALL, P.A., LEMOINE, N.R. & KADAR, N. (1987). Proliferative index in breast carcinoma determined *in situ* by KI 67 immunostaining and its relationship to pathological and clinical variables. *J. Pathol.*, **152**, 287–295.

BLOOM, H.J.G. & RICHARDSON, W.W. (1957). Histological grading and prognosis in breast cancer. *Br. J. Cancer*, **5**, 173–183.

CATTORETTI, G., BECKER, M.H.G., KEY, G., DUCHROW, M., SCHLÜTER, C., GALLE, J. & GERDES, J. (1992). Monoclonal antibodies against recombinant parts of the Ki-67 antigen (MIB 1 and MIB 3) detect proliferating cells in microwave-processed formalin-fixed paraffin sections. *J. Pathol.*, **168**, 357–363.

DU-TOIT, R.S., LOCKER, A.P., ELLIS, I.O., ELSTON, C.W. & BLAMEY, R.W. (1990). Evaluation of the prognostic value of triple node biopsy in early breast cancer. *Br. J. Surg.*, **77**, 163–167.

FISHER, B., BAUER, M., WICKERHAM, D.L., REDMOND, C.K. & FISHER, E.R. (1983). Relation of number of positive axillary nodes to pronosis of patients with breast cancer. *Cancer*, **52**, 1551–1557.

FONTANA, D., BELLINA, M., GUBETTA, L., FASOLIS, G., ROLLE, L., SCOFFONE, C., PORPIGLIA, F., COLOMBO, M., TARABUZZI, R. & LEONARDO, E. (1992). Monoclonal antibody Ki-67 in the study of the proliferative activity of bladder carcinoma. *J. Urol.*, **148**, 1149–1151.

GASPARINI, G., POZZA, F., BEVILACQUA, P., MELI, S., BORACCHI, P., REITANO, R., SANTINI, G., MARUBINI, R. & SAINSBURY, J.R.C. (1992). Growth fraction (Ki-67) antibody determination in early breast carcinoma: histologic, clinical and prognostic correlations. *Breast*, **1**, 92–99.

GASPARINI, G., POZZA, F., MELI, S., REITANO, N., SANTINI, G. & BEVILACQUA, P. (1991). Breast cancer cinetics: immunohistochemical determination of growth fractions by monoclonal antibody Ki-67 and correlation with flow-cytometric S-phase and with some features of tumour aggressiveness. *Anticancer Res.*, **11**, 2015–2021.

GERDES, J., SCHWAB, U., LEMKE, H. & STEIN, H. (1983). Production of a mouse monoclonal antibody reactive with a human nuclear antigen associated with proliferation. *Int. J. Cancer*, **31**, 13–20.

GRANATA, G., CORADINI, V., CAPPELLETTI, V. & DI FRONZO, G. (1991). Prognostic revelance of cathepsin D versus oestrogen receptors in node negative breast cancers. *Eur. J. Cancer*, **27**, 970–972.

KALLIONIEMI, O.-P., HIETANEN, T., MATTILA, J., LEHTINEN, M. LAUSLAHTI, K. & KOIVULA, T. (1987). Aneuploid DNA content and high S phase fraction of tumour cells are related to poor prognosis in patients with primary breast cancer. *Eur. J. Cancer Clin. Oncol.*, **23**, 277–282.

LOCKER, A.P., BIRREL, K., BELL, J.A., NICHOLSON, R.I., ELSTON, C.W. & BLAMEY, R.W. (1992). Ki-67 immunoreactivity in breast carcinoma: relationships to prognostic variables and short term survival. *Eur. J. Surg. Oncol.*, **18**, 224–229.

O'REILLY, S.M., CAMPLEJOHN, R.S., BARNES, D.M., MILLIS, R.R., RUBENS, R.D. & RICHARDS, M.A. (1990). Node-negative breast cancer: prognostic subgroups defined by tumour size and flow cytometry. *J. Clin. Oncol.*, **8**, 2040–2046.

PORSCHEN, R., KRIEGEL, A., LANGEN, C., CLASSEN, S., HILSE, M., LOHE, B., HENGELS, K.J. & BORCHARD, F. (1991). Assessment of proliferative activity in carcinomas of the human alimentary tract by Ki-67 immunostaining. *Int. J. Cancer*, **47**, 686–691.

SILVESTRINI, R., SANFILIPPO, O. & TEDESCO, G. (1974). Kinetics of human mammary carcinomas and their correlation with the cancer and host characteristics. *Cancer*, **34**, 1252–1258.

SLAMON, D.J. & CLARK, D.J. (1988). Amplification of c-erb-2 and aggressive human breast tumors. *Science*, **240**, 1795–1798.

THOMPSON, S.J., MELLON, K., CHARLTON, R.G., MARSH, C., ROBINSON, M. & NEAL, D.E. (1992). P53 and Ki-67 immunoreactivity in human prostate cancer and benign hyperplasia. *Br. J. Urol.*, **69**, 609–613.

TNM ATLAS (1992). Third edition, 2nd Revision pp. 185–195. Springer-Verlag: Berlin.

TOI, M., OSAKI, Y., YAMADA, H. & TOGE, T. (1991). Epidermal growth factor receptor expression as a prognostic indicator in breast cancer. *Eur. J. Cancer*, **27**, 977–980.

WEIKEL, W., BECK, T., MITZE, M. & KNAPSTEIN, P.G. (1991). Immunohistochemical evaluation of growth fractions in human breast cancers using monoclonal antibody Ki-67. *Breast Cancer Res. Treat.*, **18**, 149–154.


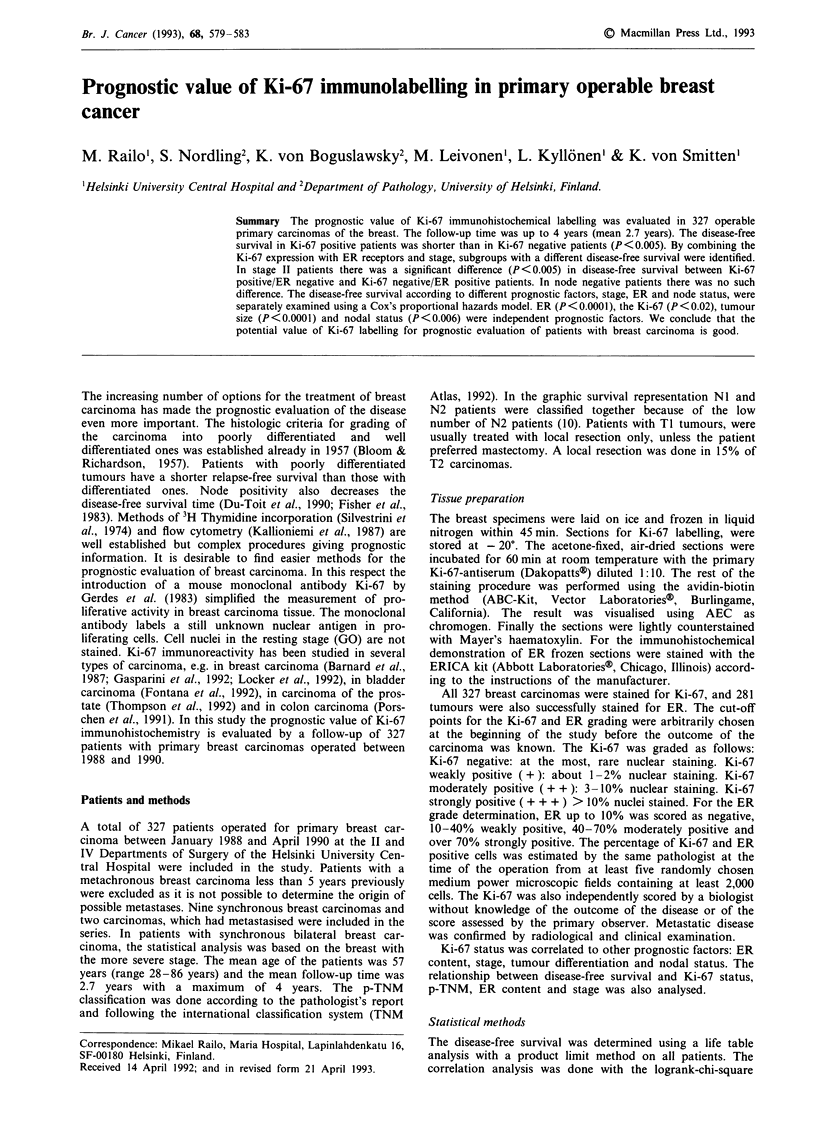

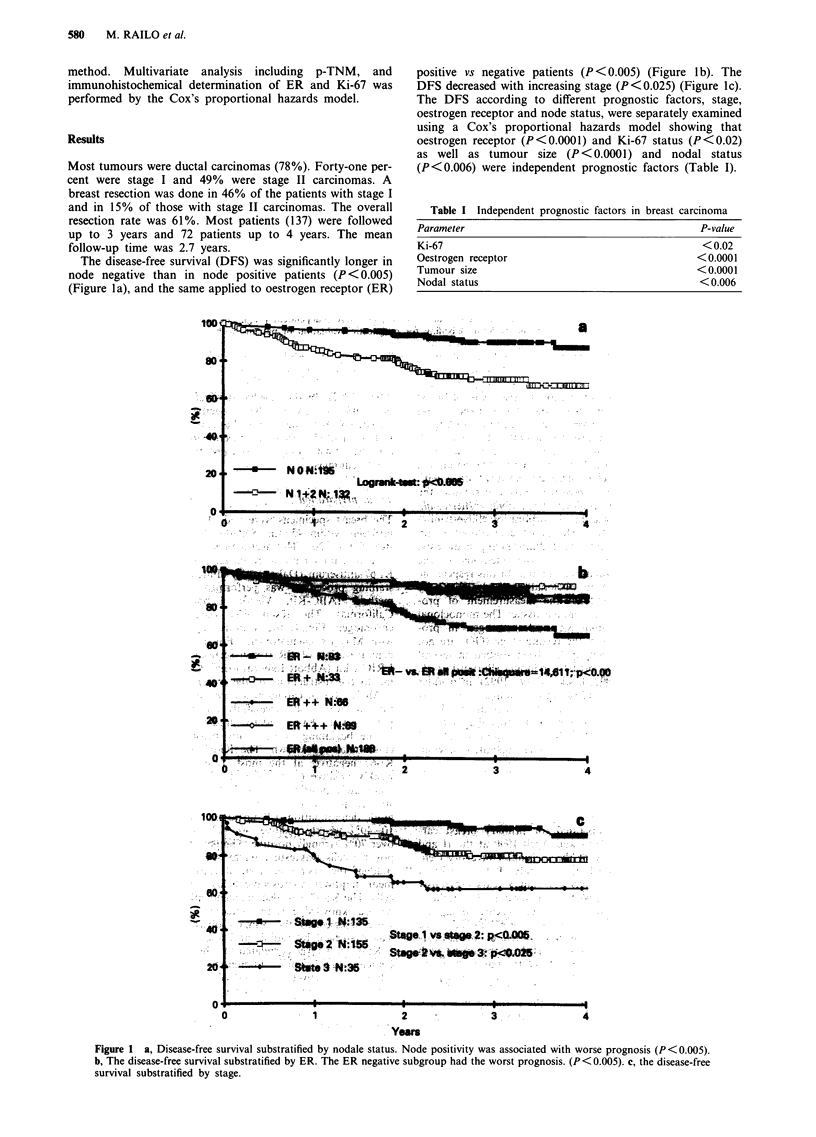

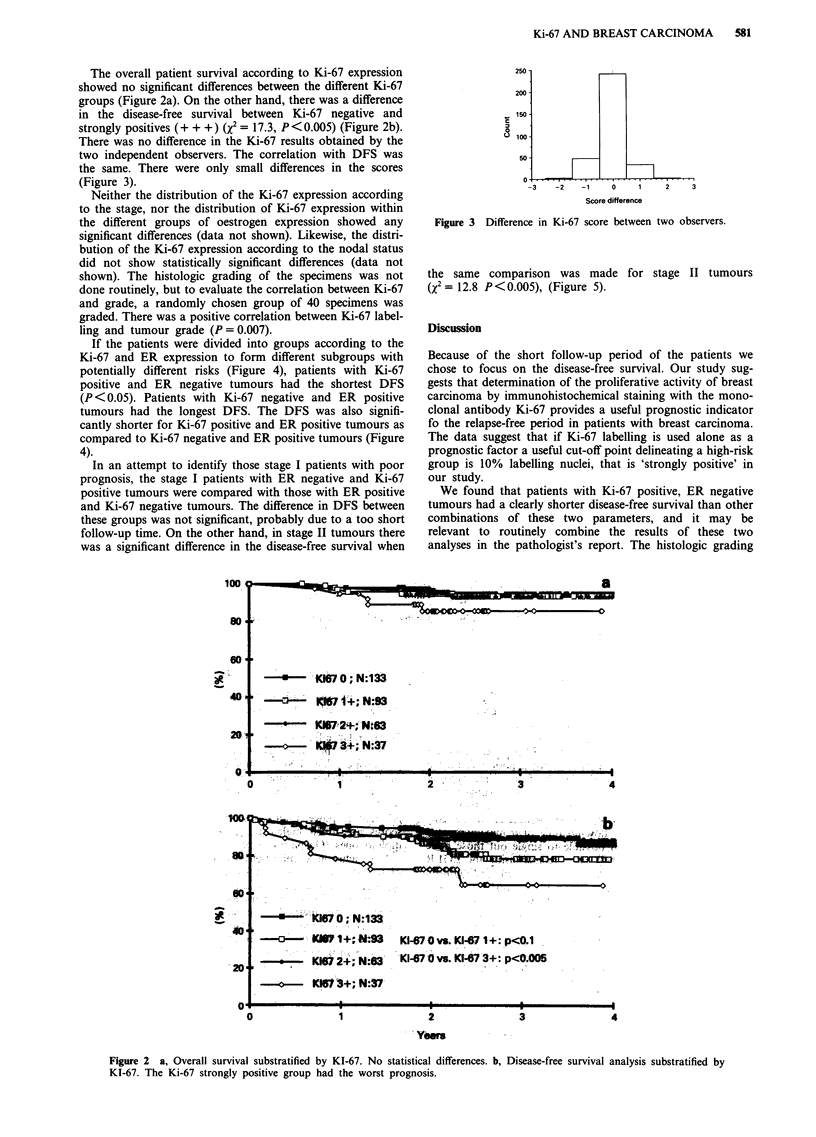

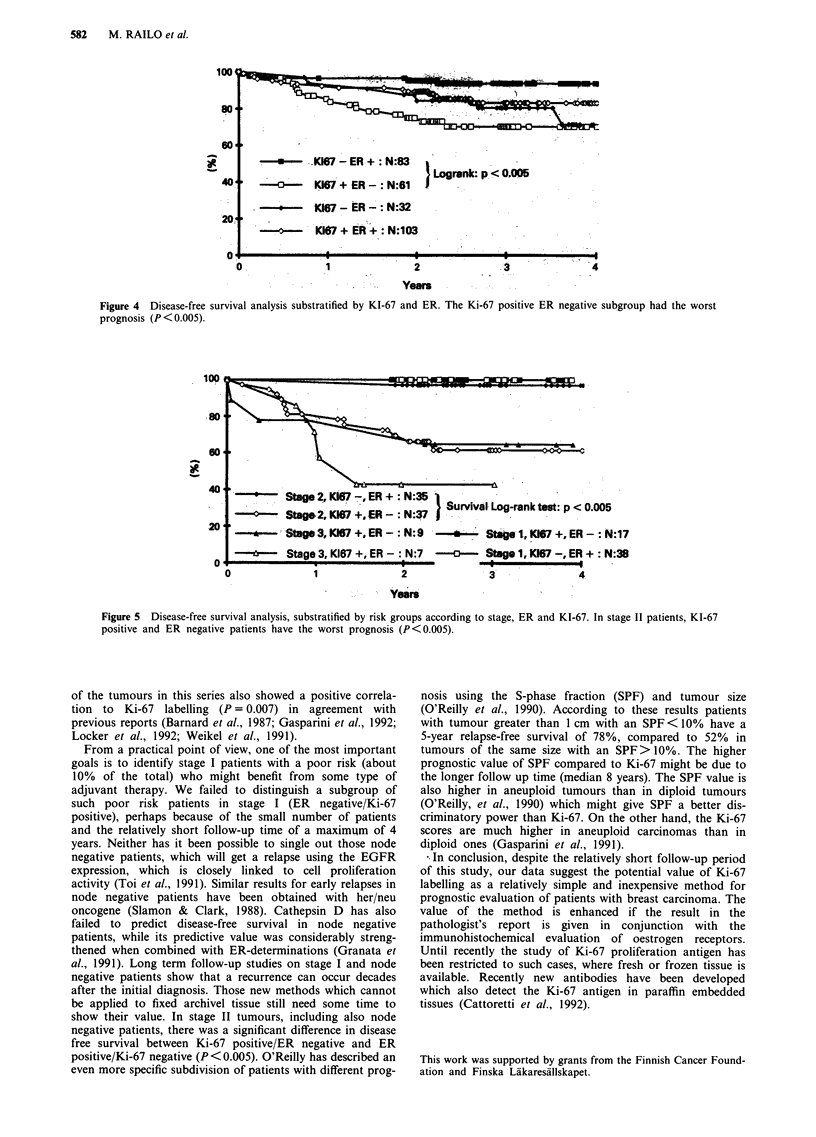

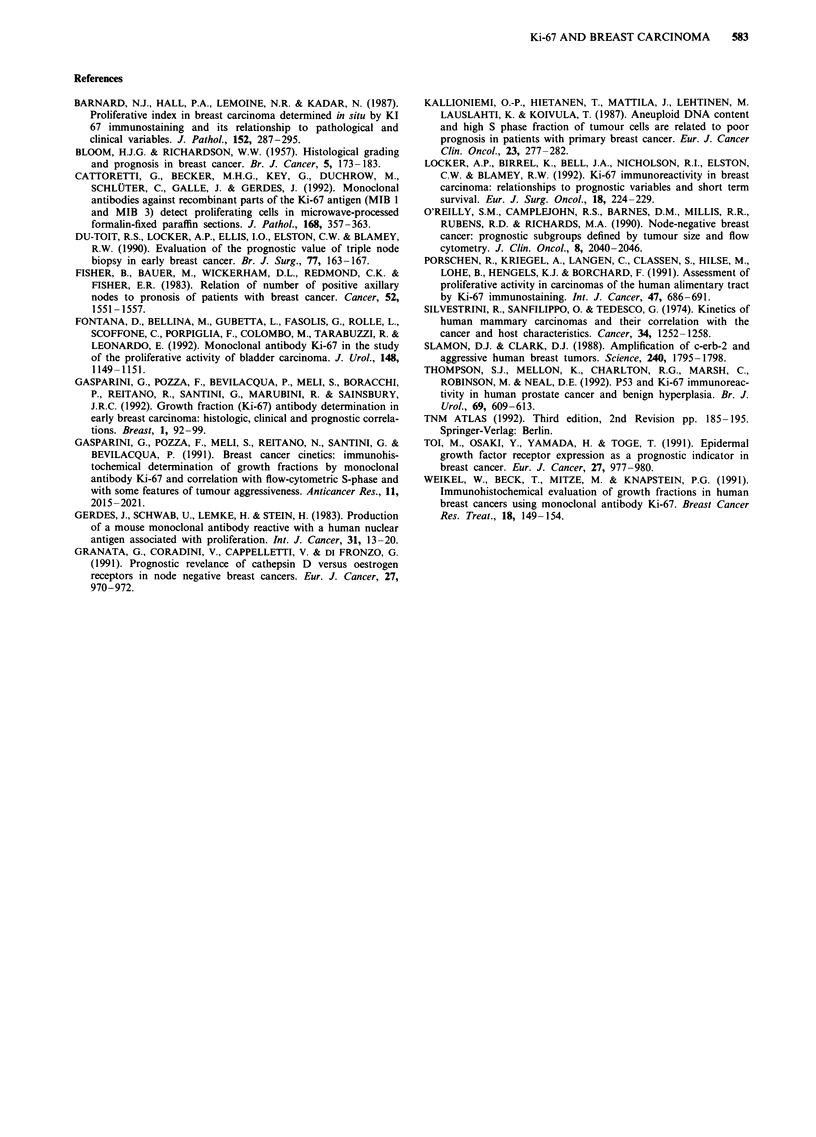


## References

[OCR_00551] Barnard N. J., Hall P. A., Lemoine N. R., Kadar N. (1987). Proliferative index in breast carcinoma determined in situ by Ki67 immunostaining and its relationship to clinical and pathological variables.. J Pathol.

[OCR_00561] Cattoretti G., Becker M. H., Key G., Duchrow M., Schlüter C., Galle J., Gerdes J. (1992). Monoclonal antibodies against recombinant parts of the Ki-67 antigen (MIB 1 and MIB 3) detect proliferating cells in microwave-processed formalin-fixed paraffin sections.. J Pathol.

[OCR_00568] Du Toit R. S., Locker A. P., Ellis I. O., Elston C. W., Blamey R. W. (1990). Evaluation of the prognostic value of triple node biopsy in early breast cancer.. Br J Surg.

[OCR_00573] Fisher B., Bauer M., Wickerham D. L., Redmond C. K., Fisher E. R., Cruz A. B., Foster R., Gardner B., Lerner H., Margolese R. (1983). Relation of number of positive axillary nodes to the prognosis of patients with primary breast cancer. An NSABP update.. Cancer.

[OCR_00579] Fontana D., Bellina M., Gubetta L., Fasolis G., Rolle L., Scoffone C., Porpiglia F., Colombo M., Tarabuzzi R., Leonardo E. (1992). Monoclonal antibody Ki-67 in the study of the proliferative activity of bladder carcinoma.. J Urol.

[OCR_00593] Gasparini G., Pozza F., Meli S., Reitano M., Santini G., Bevilacqua P. (1991). Breast cancer cell kinetics: immunocytochemical determination of growth fractions by monoclonal antibody Ki-67 and correlation with flow cytometric S-phase and with some features of tumor aggressiveness.. Anticancer Res.

[OCR_00601] Gerdes J., Schwab U., Lemke H., Stein H. (1983). Production of a mouse monoclonal antibody reactive with a human nuclear antigen associated with cell proliferation.. Int J Cancer.

[OCR_00605] Granata G., Coradini D., Cappelletti V., Di Fronzo G. (1991). Prognostic relevance of cathepsin D versus oestrogen receptors in node negative breast cancers.. Eur J Cancer.

[OCR_00613] Kallioniemi O. P., Hietanen T., Mattila J., Lehtinen M., Lauslahti K., Koivula T. (1987). Aneuploid DNA content and high S-phase fraction of tumour cells are related to poor prognosis in patients with primary breast cancer.. Eur J Cancer Clin Oncol.

[OCR_00618] Locker A. P., Birrell K., Bell J. A., Nicholson R. I., Elston C. W., Blamey R. W., Ellis I. O. (1992). Ki67 immunoreactivity in breast carcinoma: relationships to prognostic variables and short term survival.. Eur J Surg Oncol.

[OCR_00624] O'Reilly S. M., Camplejohn R. S., Barnes D. M., Millis R. R., Rubens R. D., Richards M. A. (1990). Node-negative breast cancer: prognostic subgroups defined by tumor size and flow cytometry.. J Clin Oncol.

[OCR_00630] Porschen R., Kriegel A., Langen C., Classen S., Hilse M., Lohe B., Hengels K. J., Borchard F. (1991). Assessment of proliferative activity in carcinomas of the human alimentary tract by Ki-67 immunostaining.. Int J Cancer.

[OCR_00636] Silvestrini R., Sanfilippo O., Tedesco G. (1974). Kinetics of human mammary carcinomas and their correlation with the cancer and the host characteristics.. Cancer.

[OCR_00641] Slamon D. J., Clark G. M. (1988). Amplification of c-erbB-2 and aggressive human breast tumors?. Science.

[OCR_00645] Thompson S. J., Mellon K., Charlton R. G., Marsh C., Robinson M., Neal D. E. (1992). P53 and Ki-67 immunoreactivity in human prostate cancer and benign hyperplasia.. Br J Urol.

[OCR_00655] Toi M., Osaki A., Yamada H., Toge T. (1991). Epidermal growth factor receptor expression as a prognostic indicator in breast cancer.. Eur J Cancer.

[OCR_00660] Weikel W., Beck T., Mitze M., Knapstein P. G. (1991). Immunohistochemical evaluation of growth fractions in human breast cancers using monoclonal antibody Ki-67.. Breast Cancer Res Treat.

